# Rehabilitation of Marine Turtles and Welfare Improvement by Application of Environmental Enrichment Strategies

**DOI:** 10.3390/ani12030282

**Published:** 2022-01-24

**Authors:** Cesar Marcial Escobedo-Bonilla, Noelia Maria Quiros-Rojas, Esteban Rudín-Salazar

**Affiliations:** 1 Laboratory of Pathology and Molecular Diagnostics, Department of Aquaculture, CIIDIR-Sinaloa, Instituto Politécnico Nacional, Guasave C.P. 81101, Mexico; 2 Escuela de Medicina y Cirugia Veterinaria, Facultad de Ciencias Agropecuarias, Sede Atenas, Universidad Tecnica Nacional, Atenas C.P. 20501, Costa Rica; noequiros2894@gmail.com; 3 Centro de Rescate y Rehabilitacion de Animales Marinos (CRRAM), Parque Marino del Pacífico, Puntarenas C.P. 60101, Costa Rica; veterinariarudinysalazar@hotmail.com

**Keywords:** sea turtles, conservation, rescue, rehabilitation, welfare, environmental enrichment

## Abstract

**Simple Summary:**

Sea turtles are vital members of the marine ecosystem since they contribute to keeping balance in such environments. Unfortunately, they are endangered species whose absence may be detrimental. Conservation efforts aim to preserve sea turtles both at the population and individual levels. Rescue and rehabilitation aim to reduce individual morbidity and mortality as a result of injuries mainly caused by interactions with humans, in order to preserve their genetic diversity and help maintain and/or increase their population size. Environmental enrichment (EE) is a set of methodologies aimed to improve animal welfare during captivity/rehabilitation. This review presents successful cases of sea turtle environmental enrichment and its applications to improve their welfare in captivity and to increase their fitness prior to release into the wild. EE is a valuable tool that enhances welfare during the captivity and/or rehabilitation of sea turtles and improves their chances of survival and reintegration back into wild populations upon release. EE may be adopted in rescue and rehabilitation facilities around the world to improve individual survival and help boost conservation efforts.

**Abstract:**

Sea turtles perform various ecological services in several marine environments and are considered architects of the marine landscape. At present, they are endangered species due to anthropogenic threats, pollution and degradation of marine habitats. These impacts make it urgent to increase protection and conservation efforts. Protective actions include the rescue and rehabilitation of injured individuals as a result of their interactions with humans and other threats. Environmental enrichment (EE) is a series of techniques and methods aimed to improve the welfare of animals in captivity and/or under rehabilitation. It uses external stimuli to enhance their psychological and physiological wellbeing to promote natural abilities and behaviors. These may increase the survival chances of rehabilitated animals upon release in the wild. This review presents data of studies where EE has been applied during the rehabilitation processes of different species of sea turtles, and its effect on welfare improvement during captivity/rehabilitation and on survival after release into nature. Technologies such as satellite tags are an important means to determine rehabilitation success and survival of injured individuals from endangered species after release into the wild, as they allow tracking and monitoring of such individuals, and determine their location in areas used by their natural populations for feeding or breeding.

## 1. Introduction

Sea turtles are vital in maintaining the health and balance of various ecosystems in the marine and estuarine environments [1,2,3,4]. These reptiles are also valuable since they are regarded as sentinel and keystone species and even used as flagship species, leading many of the conservation and restoration efforts of marine ecosystems worldwide [1,2,3,4,5,6].

At present, most of these species are globally threatened by extinction, according to Appendix I of the Convention on International Trade in Endangered Species of Flora or Fauna (CITES Convention), and also the World Conservation Union (IUCN) [7,8,9,10]. The Leatherback (*Dermochelys coriacea*), Hawksbill (*Eretmochelys imbricata*) and Kemp’s ridley (*Lepidochelys kempii*) sea turtles are classified as “Critically Endangered”. This category describes species that have sustained “an observed, estimated, inferred or suspected reduction of at least 80% over the last 10 years or three generations, whichever is the longer”. The Green (*Chelonia mydas*) and Loggerhead (*Caretta caretta*) sea turtles are in the category “Endangered” since they have shown “an observed, estimated, inferred or suspected population reduction of at least 50% over the last 10 years or three generations, whichever is the longer”. In contrast, the Olive ridley (*Lepidochelys olivacea*) sea turtle is classified as “Vulnerable”, since it has shown “an observed, estimated, inferred or suspected population size reduction of ≥30% over the last 10 years or three generations, whichever is the longer” [11,12].

Threats to marine turtle populations are mainly anthropogenic and include pollution of the marine environment, ingestion of plastic debris, fishery by-catch, harvesting adults and egg poaching, injuries by contact with fishing gear and boats, loss of habitat, entanglement and more [13,14]. Other threats are of natural origins, such as blooms of toxic algae, cold stunning, climate change, parasitism and infectious disease [1,2,4,14,15,16,17,18,19,20,21,22].

Actions aimed to curb the threat of extinction include urgent and coordinated multinational interventions in: (i) protecting all nesting beaches and the establishment of marine protected areas; (ii) reducing fisheries by-catch by at-sea and coastal fisheries through the implementation of turtle excluder devices; (iii) addressing Pan-Pacific policy actions; and (iv) supporting the sustainability of the traditional use of sea turtles [14,23].

Nonetheless, in certain specific areas, sea turtle populations are growing as a result of the success of various conservation efforts. In North Carolina, USA, accidental by-catch of sea turtles by inland or estuarine fisheries is an occurrence. A time-series analysis was carried out to assess the population size of the endangered Kemp’s ridley and green turtle species [24]. By-catch by the estuarine gillnet fishery increased by 318% and 676%, respectively, when compared with reports of catches per trip from 2001–2005 and 2012–2016. The gillnet fishery has shown reductions in fish catches with time, probably due to the closure of fishing areas after a certain number of turtles are caught, thus restricting the number of trips and reducing fish harvest [24].

Also, some reports of specific successful sea turtle nesting sites (rookeries) have occurred in recent years. These include data of long-term increases in the abundance of females and their nest numbers. For example, the Hawaiian green turtle subpopulation was listed in 2012 as being of “least concern”, as a consequence of a long-term increase in the size of this population [14]. A time-series analysis of regional management units (RMUs, discrete groups of nesting sites in areas distinct from one another based on genetics, distribution, movement and demography), showed seventeen RMUs with significant abundance trends, twelve increasing and five decreasing. The increasing abundance trends were observed for one of one RMUs for hawksbill and Kemp’s ridley turtles, respectively; three out of three RMUs for loggerhead, four out of five RMUs for the green turtle, two out of three RMUs for olive ridley, one out of three RMUs for leatherback turtles and zero out of one for flatback turtles. In contrast, RMUs with decreasing abundance were found for leatherback turtles in RMUs 55 and 56 from the Eastern and Western Pacific, respectively, and the RMU60 in the Southwest Pacific for the flatback turtle [14].

Another important action for the conservation of endangered species is rescue and rehabilitation, which contributes to maintaining wild populations by releasing rehabilitated sea turtles back to their natural environment. Rescue and rehabilitation centers also promote conservation through education of the public and research [25,26,27]. Rehabilitation requires the implementation of environmental enrichment techniques in order to promote natural behaviors and enhance innate skills needed by the sea turtles to return to their natural populations. The present review aims to show the effect of environmental enrichment on rehabilitation welfare and the efficacy of survival upon sea turtles released into the natural environment.

## 2. Threats to Sea Turtles in Coastal and Marine Environments

Sea turtles endure various anthropogenic and natural threats throughout their lives and across the environments that they live in. Anthropogenic risks include entanglement and/or incidental capture in fishing gear—which causes injuries and/or mortality—as well as predation or destruction of nests and loss of nesting and marine habitats, climate change and disease [2,17,20,28].

The human impact on sea turtles, caused by the consumption of their meat and eggs or the use of their derivatives as ornaments, is a major factor threatening their survival [28]. Further, carnivorous or omnivorous sea turtle species are threatened by fisheries by-catch as they prey upon aquatic species that dwell at the bottom of shallow waters, such as crabs, shrimp and clams. Hence, turtles are often incidentally caught by trawling fisheries. Also, species of turtles that live in pelagic or neritic zones can be trapped trying to feed upon the bait of oceanic longlines or are caught by drift nets [1,17].

Recreational angler fishing is another factor impacting incidental sea turtle by-catch. In the period 2010–2015, the Mississippi Sea Turtle Stranding and Salvage Network (STSSN) reported 1073 by-catch cases, mostly juvenile Kemp´s ridleys. A survey conducted in 2013 collected information and promoted awareness and outreach to anglers about catching sea turtles. Participation was high (86%), and over 60% of anglers used J hooks for general fishing. Bait used was mainly dead shrimp (58%) and minced fish, which was very different from STSSN reports, where 60% of turtles were caught on fish and only 6% on shrimp. More than 18% of participants captured at least one sea turtle in the last year and said that almost half of them were taken to rehabilitation, 41% were released by the angler and 10% broke the line and swam out. Most of the anglers (60%) reported the capture but many were unaware they should do it [29].

Motorized watercrafts used for recreation in estuarine and marine environments pose a threat to sea turtles. In Florida, USA, between 1986 and 2014, about a third of stranded loggerhead, green and leatherback turtles had vessel-strike injuries (VSI) [30]. Kemp’s ridley and hawksbill turtles had a lower incidence of VSI (26.1% and 14.8%, respectively). The annual VSI incidence of stranded sea turtles (loggerhead, green and Kemp’s ridley) increased with the annual number of vessels registered in Florida. The occurrence of VSI was highest for adult loggerhead, green and leatherback turtles since these were the reproductively active individuals most susceptible to these harms. Necropsies of 194 stranded sea turtles with a VSI showed that these injuries were the most likely cause of death in over 92.8% of cases. The mean annual amount of sea turtles dead by this threat may range from 160 up to 2300 individuals, depending on the species. The risk of VSI was associated with inlets or passes, marinas or navigable waterways [30].

Pollution of the marine environment by anthropogenic garbage is a major threat to sea turtles, particularly the presence of plastic bags and other items, since these may be confounded by the jellyfish-eating species along with their natural prey [1,20,22]. Other types of pollution include oil spilling, heavy metals and other toxins, which can be damaging or fatal to sea turtles [19,20]. Oil spills and chronic pollution by tanker wastes in the marine environment can damage habitats such as seagrass beds and coral reefs by inducing declines in intertidal and subtidal reef fauna diversity due to mortality, seagrass biomass and sublethal effects such as reduced reproduction rates in these ecosystems [22].

Natural predation is another threat faced by sea turtles. This may be an important element acting on the structure of their populations in many regions. Sharks are the most frequent natural predators of adult marine turtles worldwide [2,21], but orcas can also prey upon them [18,19]. In nesting zones, natural predators include crocodiles and felines such as jaguars, panthers and tigers. Hatchlings are predated in beaches and coastal shores by crabs, birds, fish and mammals [4,18,19]. Predation may also shape turtle behavior and population density. Nonetheless, this is not the only aspect that influences changes in these features, since movement, habitat use patterns, reproduction and foraging also play a role in such issues, at least in some species [2]. Sea turtles are part of the trophic food web in marine ecosystems since they play a role as consumers of lower food strata and are hunted by some predators higher up on the chain [21]. Other ecological functions are as keystone species since they are fundamental to keeping the balance in coral reefs and seagrass bed ecosystems [7], and as sentinel species, as they help to determine the health of coastal environments [2].

Environmental conditions promote or hinder the movements of juvenile sea turtles to nursery habitats and may be greatly influential on population abundance and survival of hatchlings and juveniles. Major disturbances, such as hurricanes, can impact sea turtle dispersal and survival. An ocean circulation model was developed to simulate seasonal and annual variations in the post-hatchling dispersal of Kemp’s ridley sea turtles [31]. Cohorts (*n* = 24) of young-of-the-year Kemp’s ridley sea turtles dispersing from three primary nesting areas in the Western Gulf of Mexico were used to describe transport variability during the main hatching season and across years. Results suggested that hurricane frequency and intensity may influence sea turtle survival and growth rates from different nesting sites and hatchling cohorts. This may be either positive, improving survival by increasing retention in optimal pelagic habitat, or negative, decreasing survival by pushing hatchlings into dangerous shallow habitats [31].

Ocean currents are another factor influencing biological aspects such as reproduction timing, the location of breeding sites and variations of spatiotemporal recruitment. They may also shape the gene flow magnitude and direction among populations, colonization and speciation at larger timescales [32]. Hatchling migration is a critical period in the sea turtle life cycle—it may sustain mortalities up to 85% depending upon beach geography and conditions, predator density and nearby ocean currents. Regional variations in population size may depend on differences in ocean currents and how they aid hatchling transport offshore. This oceanic transport may also influence the distribution of juvenile sea turtles and the subsequent selection of foraging grounds at the adult stage. A study was conducted to investigate the spatiotemporal variability of oceanic transport of sea turtle hatchlings from 67 nesting beaches across the world. Data of 25 years of ocean circulation models were paired with virtual particle tracking software to simulate the ocean-driven transport of non-swimming hatchling sea turtles. The simulation showed that at 30 d of hatchling transport from the nesting beach, the mean range of transport distance varied between 67 ± 64.7 (standard deviation [sd]) km (Chiriqui, Panama) to 817 ± 254.5 (sd) km (Galapagos Islands, Ecuador). The global mean transport distance at 30 d was 307 ± 151.7 (sd) km. Distance variation was higher between sites, but inter-annual variations were also observed and depended on current shifts at specific locations for any given year ([32]. Transport distance was greatest in the tropical latitudes. Ocean region, coast type and latitude all played important roles in ocean driven sea turtle hatchling transport. Clear differences in transport distance across ocean regions were determined. Regional differences in hatchling transport and the status of adult sea turtles could improve conservation assessments for specific populations [32].

Climate change is a major threat to marine turtles because they are dependent on atmospheric and water temperature since it plays a role in the sex determination of embryos, their long-life cycles—including their long age-to-maturity—and their migratory nature [33]. These animals have survived past climate changes, including glacial and warming periods; hence, they have some ability to adapt to drastic climate shifts. However, the present steep increases in atmospheric greenhouse gas concentrations and its association to fast temperature increase may overcome their adaptation ability to temperature changes [33]. The impacts of climate change and its consequences on marine turtles may be complex and mostly negative. The most obvious effect of climate change would be a shift in hatchling sex ratios towards females, diminishing the rate of males. Its effect cannot be exactly determined but it may reduce the breeding rates of the species, although some populations may be resilient to warming if female biases remain within levels where population success is not impaired. Another effect would be the rising sea levels and increased storm intensity, which may have a negative impact on turtle nesting beaches, since these events may erode or flood the existing nesting places. Additionally, extreme storms can also lead to the development of coastal lines. Alteration of wind patterns and oceanic currents will affect the migratory patterns and distribution of juvenile sea turtles in the oceans and hence their survival. Still, the migratory nature of sea turtles and their ability to move considerable distances in short periods of time would increase their resilience to climate change [33].

Climate change is also likely to impact sea turtles through changes in food availability, shifting their feeding habitats. Species associated with coral reefs are susceptible to coral bleaching, a phenomenon growing stronger and persistently around the world. This abnormality may trigger other coral diseases, causing mortality, reduced reef productivity, algae overgrowth and invasion. This effect of climate change may have more impact on some regions where man-made impacts have occurred, such as redistribution of shoreline sediments and increased water turbidity [2,33].

Stranding of moribund or dead sea turtles can provide valuable information of population trends such as age, size structure and reproductive status, diet and health. They also present useful evidence about the geographic distribution and abundance of a species and determine the main causes of declining populations exposed to anthropogenic risks [15]. In Hawaii, a survey conducted on sea turtle strandings from 1982 to 2003 reported a total of 3861 strandings comprising five sea turtle species, with the green turtle being the most abundant (97%). A total of 3732 stranded green turtles were reported, of which 75% were recorded at Oahu, where this phenomenon occurs year-round. Necropsy analyses showed that the most common causes of stranding were the tumor-forming disease fibropapillomatosis (28%), injuries caused by hook-and-line fishing gear (7%), gillnet fishing gear (5%), VSI (2.5%) and shark attacks (2.7%). Other stranding causes accounted for 5.4%, whereas the remaining 49% of strandings were by unknown causes [15]. The specific mortality rate was 88% for fibropapillomatosis, 69% for gillnet gear and 52% for hook-and-line gear. The highest probability of stranded dead green turtles occurred between 1982 until the mid-1990s, when it began to subside. This diminished risk of mortality is probably due to the reduced prevalence and severity of fibropapillomatosis, as well as the lower mortality risk due to fishing gear. Despite these risks, the Hawaiian green turtle population seems to be recovering as a result of conservation efforts committed since the late 1970s. Nonetheless, strandings due to incidental fishing have continually increased since 1982 [15].

A study carried out over a four-year period (2006–2009) on 100 stranded green turtles from southern Queensland determined the causes of stranding and morbidity through postmortem examinations. Parasitism caused by spirorchiid trematodes was the most frequent cause of mortality (41.8%), followed by gastrointestinal impaction (11.8%), microbiological infections (5.2%) and trauma (5.2%). The most common diseases were spirorchiid parasitism with associated inflammation (75%) and gastrointestinal impaction (5.1%), whereas other diseases such as cachexia, renal, digestive and respiratory malfunctions were observed at a low prevalence. Analysis of the likelihood of disease influenced by risk factors such as season, maturity and gender showed that spirorchiid infestation was more common in summer compared to other seasons, that immature turtles were more susceptible to this parasitism than mature turtles and that respiratory diseases were more likely in summer–autumn than in winter–spring. The frequency and severity of observed cases of spirorchiid infestations were highest in the brain compared with other examined organs. These findings may aid management decisions and determine the significance of green turtle survival in Queensland [16].

Likewise, a long-term (1998–2014) survey on a large population of stranded loggerhead sea turtles (*n* = 1860) admitted to the Tafira Wildlife Rehabilitation Center (TWRC) in Gran Canaria Island, Spain, was conducted to analyze the causes of stranding using specific epidemiological data to analyze the outcomes of rehabilitation [34]. Seven categories of morbidity were determined: (i) entanglement in fishing gear and/or plastics; (ii) ingestion of hooks and monofilament lines; (iii) trauma; (iv) infectious disease; (v) crude oil; (vi) other causes; and (vii) unknown/undetermined. Outcomes were calculated as euthanasia (Er), unassisted mortality (Mr) and release (Rr) rates. Time to death (Td) for euthanized and dead turtles and the length of stay for released (Tr) turtles were also determined. The most frequent morbidities were entanglement in fishing gear and/or plastics (50.81%), unknown/undetermined (20.37%) and hook ingestion (11.88%). Of the 1634 loggerhead turtles admitted alive, their final disposition was: euthanasia (Er) *n* = 55, 3.37%; unassisted mortality (Mr) *n* = 169, 10.34%; and release (Rr) *n* = 1410, 86.29%. Euthanasia (18.67%) and unassisted mortality (30.67%) were significantly higher in turtles admitted due to trauma, compared to the other causes of admission. Release was highest in animals admitted due to crude oil (93.87%) and entanglement (92.38%). The median length of stay for released turtles varied from 12 d (unknown) to 70 d (trauma). This was the first large-scale epidemiological study on causes of stranding and mortality of the Eastern Atlantic loggerhead population, showing anthropogenic factors (71.72%) as the main cause of stranding and mortality [34].

Injuries and diseases are often a consequence of human activities, causing trauma and infections, impaired swimming and feeding due to intussusception and/or intestine rupture and secondary infection [20]. Pathologies found in injured sea turtles include lung edema and emphysema, net marks on the skin, muscle necrosis and hemorrhage in the coelom. Incidental fishing can cause decompression sickness, inducing a coma, hyperactive or progressive neurological symptoms or death. Ultrasound and post-mortem analyses in injured sea turtles showed damage such as gas bubbles in the lungs, liver, kidney, spleen, heart and major vessels, perivascular edema and hemorrhage in different tissues [20]. Trauma caused by collisions may result in crushed tissues, hemorrhage in the head, carapace and plastron and even flipper amputation. These injuries can cause weakness, disorientation and secondary infectious diseases in the lungs and kidneys, and often result in death [20].

Environmental changes such as alterations in water temperature and resource availability may contribute to the more frequent appearance of emerging diseases, expanding host ranges and new signs of disease [16]. As an example, the virus-caused disease fibropapillomatosis (FP), which develops tumors in external and internal soft tissues, was first identified in green turtles in Florida in 1938. It emerged as a global epidemic in the 1980s, affecting most sea turtle species, while other manifestations of the disease, such as corneal involvement, were not reported until the 1990s [16].

Viral diseases in sea turtles include fibropapillomatosis (FP), which consists of multifocal cutaneous or visceral tumors in juvenile or adult animals. This neoplasia is benign, but depending on its size, number and location, it can cause problems such as impaired vision, diving and feeding [20,35,36,37]. The FP is distributed worldwide among sea turtles. The chelonid herpesvirus 5 (ChHV5) has been detected in tumor tissues from different sea turtle species and regions and is recognized as the etiological agent. This pathogen, along with certain environmental factors such as high pollution levels and high-water temperature, may trigger tumor formation [20,35,36,37]. Other viral diseases affecting the skin include one caused by a herpes virus called papular dermatitis, or gray-patch disease, which is associated with a secondary bacterial infection in captive animals. An epidermis hyperplasia in *C. mydas*, which produces whitish small lesions with severe hyperkeratosis and intranuclear inclusion bodies, is called *Chelonia mydas* papillomavirus (CmPV) [20,38].

Epibionts living on free-range or captive sea turtles, such as leeches and barnacles, cause contact diseases such as ulcerative dermatitis and fibrosis because of the trauma caused by biting or fixing into them. The severity of lesions depends on the distribution and number of epibionts, sometimes leading to anemia, extensive dermatitis and occasional secondary bacterial or fungal infections [20,38].

Sea turtles may harbor several bacterial species that may be opportunistic pathogens to many vertebrate species, and/or cause disease to humans (zoonotic). These include the genera *Mycobacterium*, *Salmonella*, *Vibrio* and *Chlamydia*, among many other species of clinical concern [39,40]. Degenerative processes such as abscesses, hepatitis or septicemia have been observed as being caused by different bacteria genera such as *Aeromonas*, *Bacteroides*, *Citrobacter*, *Enterobacter*, *Escherichia*, *Mycobacterium*, *Pasteurella*, *Proteus*, *Pseudomonas*, *Salmonella*, *Serratia* or *Staphylococcus*. Different lesions such as stomatitis, carapace ulcerations, focal infections in limbs and pneumonia were recorded from by-catch victims [38].

## 3. The Aim of Rescue and Rehabilitation Centers and Their Role in Sea Turtle Conservation

Rehabilitation, as the aim of a rescue/rehabilitation center, involves providing immediate temporary care as necessary to sick and/or injured wild animals in order to save their lives and allow them to regain health and normal behavior. The desired end will always be that the animals are eventually released back to their natural environment at the earliest possible time, able to fulfill their ecological roles [27,41,42,43].

Rehabilitation has become a fundamental action in the conservation of threatened or endangered species, and the preservation of biodiversity in many countries. This activity is instrumental in raising awareness of animal welfare issues at various scales. Rehabilitation centers are at the forefront of outreach efforts contributing to public awareness and education on conservation threats, as well as their mitigation, and they may promote long-term changes for the benefit of endangered species [27].

Many injuries caused by anthropogenic agents are potentially fatal to individual sea turtles. Rehabilitation can save injured animals that normally would die if left unattended. For example, severe wounds and amputations caused by entanglement in discarded monofilament lines may induce death without treatment. Thus, individual rescue and rehabilitation can be substantial to small, threatened populations, such as hawksbill turtles in the Arabian Gulf, due to their low breeding numbers and low genetic variability. This region is considered a “High Risk, High Threat” RMU for hawksbill and olive ridley turtles, making it an ideal location to develop and implement rehabilitation programs [27].

The activities comprised in these conservation programs include caring for injured or sick animals, with the aid of trained veterinarians and specialized facilities and equipment to perform diagnosis, clinical treatments and, if needed, surgical techniques. Such care involves nursing in captivity for periods of several weeks or, in some cases, years before they can be released [42]. A measure of rescue and rehabilitation success is the release of turtles back into the wild, although this contribution to sea turtle recovery may be small compared to other conservation activities [44,45].

Despite extensive care, some individuals are considered unfit for release. If such animals are not euthanized, they are often placed in permanent homes in zoos or aquaria. Each year, the number of successful rehabilitation cases and sea turtles released is very small compared to the size of the entire population [42]. Nonetheless, the information gained through rescue and rehabilitation on elucidating pathways to disease, testing treatments and assigning causes of morbidity and mortality to stranded turtles is highly valuable. Moreover, these care facilities and aquaria also play a number of important roles of direct benefit to sea turtles. These include research, environmental education and the chance of changing human behavior towards the conservation of sea turtles and the marine environment through outreach and public awareness, although these roles are not well documented [42,45].

Although rehabilitation is a major action towards the conservation of sea turtles, some aspects of it have been mentioned as drawbacks. These include the costs and their actual effect on the conservation efforts. The costs of sea turtle rehabilitation involve high economic resources due to requirements to get suitable facilities, trained staff and finance commitments. The rehabilitation costs per animal are highly variable depending on the place and the individual interventions, although it may roughly be around several thousand US dollars. Financing sources are also varied and include regular government budget, public, philanthropic and/or corporate donations and trusts and additional funding from visitor entrance fees into the facilities to observe the animals [42,44]. Although it is costly for turtles to be rehabilitated, it remains an important part of conservation as it can be a tool to educate the public about threats to sea turtle survival [42].

The contribution of rehabilitation to conservation efforts is one of the main arguments against, as occasionally it is regarded as a waste of time by numerically minded conservationists who may view the effort as inconsequential in the larger scheme of things [41]. Other considerations against rehabilitation are those stating that most animals kept captive for years may be unable to incorporate again into their populations. These may show chronic stress and immunosuppression, along with the presence of microorganisms or parasites that may weaken them or make them a vector to spread such pathogens into the natural populations, putting them at risk of novel diseases and mortalities or causing “genetic pollution” [10,40]. Another argument against the release of long-time captive animals is that they may become used to human presence, conditioned behavior and monostrophic diets. Moreover, little knowledge exists on whether rehabilitated sea turtles have successfully re-adapted, particularly individuals that required long and complicated treatment [42]. Both ethological deviations and alterations derived from unsuitable diets constitute a drawback for readaptation and survival in the wild [40]. Despite these arguments, endangered and threatened species such as sea turtles need every individual, especially those close to breeding age that are released back to the wild, in order to improve their chances towards species survival [41].

The importance of rehabilitation facilities for conservation is shown in the long-term survey conducted on a large population of loggerhead sea turtles rehabilitated at the Tafira Wildlife Rehabilitation Center (TWRC) in Gran Canaria Island, Spain [34]. A high overall percentage (86.29%) of rehabilitated sea turtles were released back to their natural environment. In order to allow comparisons between rehabilitation centers worldwide, they suggest including in the outcome of sea turtle rehabilitation processes the causes of admission to rehabilitation, the final disposition rates, the time to death (Td) for euthanized and dead turtles and the length of stay for released turtles (Tr) [34].

Satellite-tracking data on the movements and survival rates of rehabilitated sea turtles showed similar behavior as wild (control) animals. Upon release into their natural environments, most rehabilitated turtles displayed almost normal dispersal behavior, searching for foraging or breeding sites, or in turtles with amputated flippers, their swimming ability was not reduced compared to non-amputated animals [27].

A study carried out under the Dubai Turtle Rehabilitation Project (DTRP) in the Arabian Gulf establishes the effect of rehabilitation of sea turtle species admitted with different ailments, including flipper amputation, on survival upon release into the environment. Comparisons were made on movement characteristics, survival and ecology between the amputee and non-amputee individuals of the same species [27]. Satellite tags were used to assess turtle rehabilitation success as survival in the wild after release. The study involved 26 sea turtles entering the DTRP between March 2012 and January 2018 with conditions at admission such as cold stunning (*n* = 3), VSI traumas (*n* = 4), general debilitation (*n* = 7) and infections (*n* = 6). Another six sea turtles were admitted with one front flipper amputated by injury, or a flipper amputated by a veterinarian due to entanglement damage. Rehabilitated sea turtles were hawksbill (*n* = 12), loggerhead (*n* = 11), green (*n* = 2) and olive ridley (*n* = 1) [27]. Sea turtles were classified as either juvenile, sub-adult or adult based on species-specific curved carapace lengths. All adult sea turtles were female, while juveniles and subadults were undetermined. Sea turtles were released between May 2012 and May 2018, after rehabilitation which lasted between 89 and 817 d (mean 353 ± 237 d). Post-release satellite tracking of sea turtle movements and survival were studied for 8 to 837 d (mean 155 ± 95 d). Tag data suggested that three sea turtles died within four days after release, one after twenty-seven days and another after a hundred and ninety-two days. The causes of death were anthropogenic factors independent of their pre-rehabilitation conditions. Loggerhead turtles showed the highest dispersal, with 80% crossing an international border. Hawksbill turtles displayed higher post-release residency, with 66% staying in UAE territorial waters. Amputee turtles showed similar movement as non-amputee animals of the same species. Loggerhead turtles travelled faster (15.3 ± 8 km/d) than hawksbill turtles (9 ± 7 km/d). This study showed that rehabilitated sea turtles, including amputees, can successfully survive in the wild after release for at least up to one year, and it supports the release of sea turtles healing from major injuries such as amputations [27]

## 4. Environmental Enrichment (EE), Meaning and Usefulness

Environmental enrichment (EE) is a systematic, scientific approach to understanding and providing for the psychological and behavioral needs of captive animals or those undergoing rehabilitation in captivity [46,47,48,49,50]. This is a discipline based on the fields of ethology, psychology and animal science, intended to provide novel ways of environmental welfare for captive animals [46]. The EE identifies and provides the environmental stimuli needed or provides choices in the environment to increase behavioral opportunities for optimal psychological and physiological wellbeing [46,49,50]. The EE uses tools such as inanimate objects, social agents (conspecifics or contraspecifics) or sensory material (scent trail or alarm-call playback) to encourage the performance of normal or natural behavior patterns [46]. The use of EE increases the biological relevance of an enclosure and enhances the welfare of captive species by reducing the performance of abnormal repetitive behavior or stereotypies or correcting other deficiencies. It is increasingly being used in a proactive manner to create a rich, stimulating environment [47,49,50,51]. Providing stimuli in the environment is necessary for developing the expression of the appropriate behavioral and mental activities of a species in a monotonous environment. The goals of environmental enrichment are to: (i) increase behavioral diversity; (ii) reduce the recurrence of abnormal behavior; (iii) increase the range of normal behavior patterns; (iv) increase positive utilization of the environment; and (v) increase the ability to cope with challenges in a more normal way [50]. It is important to provide the appropriate EE according to the specific biology (to the extent to which it is known) of the species under consideration [46,47]. The EE strategy needs to be well planned to achieve its goals; otherwise, it can be more damaging than beneficial [49].

### 4.1. EE as Tools against Boredom and Stereotypies

The application of EE programs represents an improved opportunity for animals to display conventional species-specific behaviors producing wellbeing in captivity or rehabilitation, and better animal management. In the beginning, these programs were primarily applied to terrestrial mammals and were rarely used in non-mammalian species such as reptiles. Characteristics of such EE programs include setting objectives based on natural and species-specific behaviors, individual animal history including constraints of captivity and/or rehabilitation. These programs also require methods to quantify and evaluate the effectiveness of the enrichment [46].

Some animals in the wild are wide-ranging and opportunistic feeders, but once in captivity, they are prone to developing abnormal behavior patterns such as pacing or stereotypic swimming. Likewise, some marine turtles in the wild exhibit similar behaviors since they migrate long distances and are strong pelagic swimmers. Hence, in captivity, they often display stereotypic swimming patterns. Many captive turtles on display in aquaria come from the wild and may have special needs because of previous injuries or diseases. Such needs must be provided within a safe and healthy environment, thus making effective EE even more challenging [48]. Due to these issues, it is difficult to develop EE programs for sea turtles in captivity/rehabilitation since their natural behavior and natural history are largely unknown, although some behaviors related to foraging, hunting, socializing and sensory biology are little known. Concerning their natural history, sea turtles are known to have a varied diet, a long lifespan and require up to 50 years to become sexually mature [48].

It is complicated to establish a relationship between the performance of stereotypic behavior and wellbeing, although it is suggested that it is not a linear relationship. The performance of stereotypies may not correspond to the current well-being because stereotypies may be an effect of previous suboptimal environments. Hence, stereotyping may be a mechanism of coping with an aversive environment. Thus, individual animals that perform stereotypies in suboptimal environments may well have better welfare than those that do not perform stereotypies in the same environment. Although stereotypies are a bit more correlated with wellbeing, a survey showed that 68% of environments causing stereotypies were associated with diminished welfare. Therefore, the meaning of stereotypies in environments such as zoos or rehabilitation centers should be taken seriously as a warning sign of potential suffering, but it should not be taken solely as an index of welfare. Stereotyping animals should be considered at high risk of suboptimal welfare [47].

The EE can be divided into different categories. These include the nutritional, physical, sensory, occupational (including training) and social [47,51]. These categories comprise eight types of enrichment: feeding, structural (enclosure rotation and/or renovation), tactile, olfactory, visual, auditory, social and human-animal [50]. These types of enrichment can be classified in more than one category and are not mutually exclusive. The end goal of enrichment should be to attempt to incorporate all of these types at some point in an enrichment plan [50]. This allows the animal multiple choices of interacting with the EE [51]. For example, a food puzzle (nutritional) where the animal must extract food from a plastic tube may also provide sensory, occupational and (indirectly) social enrichment [51].

Most enrichment programs are specifically designed to meet certain environmental and individual conditions of objectives, such as avoiding boredom (through the introduction of movable items such as food, sensory or cognition), or fixed enrichment items (such as the renovation of enclosures) [49]. To increase animal activity such as rotating from enclosures, stimulate hunting or foraging behavior (through the use of a puzzle feeder), increase time to feeding activities such as search (scatter or hide food), capture (live prey), extract (puzzle feeder), handle and process food (vegetation/browse, ice blocks with food, whole food, carcasses) and increase variability of feeding times or the number of feeding times per day [47]. Many of the enrichment items used in daily scheduled activities are movable items such as toys, puzzles, hidden food, frozen food and other different feeders. Social stimulation can also be a source of enrichment, thus providing social interactions (interspecific, intraspecific, and human-animal) for captive animals by allowing them to see or touch other animals [49]. The EE interventions have proved efficient in reducing undesirable behavior or stereotypies and have increased behavioral diversity in the performance of species-specific behaviors [52].

### 4.2. EE Drawbacks

Despite the fact that EE has been shown to improve the welfare of animals in captivity and/or under rehabilitation, some issues have been raised that could slow or complicate the interpretation of its utility. The application of EE methods will never replace other aspects of welfare such as poor enclosure design, lack of healthcare or other poor management activities. Enclosures are important for increasing the correct stimulation for the animals, but they lack the sufficient stimulating effect to provide different types of enrichment to the subjects. EE is an important aspect of positive animal welfare but cannot fix care deficiencies alone, resulting in poor welfare. Nonetheless, EE plays an essential role in the stimulation of a broad spectrum of natural behaviors [47].

An aspect that complicates the evaluation of the efficacy of EE methods is the experimental design of EE applications. When different EEs are simultaneously applied to produce positive effects in the subjects, this setup does not let determine which of the EE forms was the most influential in producing the positive result [47]. Another issue is the report of only successful EE strategies for the situations of their subjects. This fact also limits the ability to determine which EE methods were beneficial and which ones were not. Designing studies testing the effect of different EE forms, including those that may be thought not to influence stereotypies in the animals, would help to determine whether all EEs work equally well and why [47].

It is possible that EE may not correct certain stereotypies that have begun as a result of different situations (increased crowd size, onset of breeding season, background noise such as traffic or construction or any combination of these and other factors) since they were not designed to mitigate the underlying causes driving such stereotypies [50]. Other situations in which EEs may not work include stereotypies that probably developed from situations of unavoidable stress or fear, poor environments, or the inability to fulfill species-appropriate activities. Some stereotypies may not reflect current conditions but remain from past experiences [50].

The cost of EE programs is another drawback since most settings have an insufficient budget to apply the actions and programs planned by the administration. Financial support from the government to zoos and other settings where animals are kept captive for exhibition or rehabilitation is also scarce, making it difficult to provide such programs. An alternative to reduce costs includes the use of recyclable materials to build EE items or to collect food items in natural areas or wildlife reserves, and the possibility to see or be close to other conspecifics are cheap and effective EE methods for many species [49].

## 5. Case Studies of EE in Sea Turtles

The first case is the report of EE in four sea turtles—three loggerheads (*Caretta caretta*) and one blind green turtle (*Chelonia mydas*) [48]. Each of these turtles was provided with four different enrichment devices (EDs). The three loggerhead turtles were provided with (i) one plastic water cooler jug and (ii) two sets of polyvinyl chloride (PVC) pipes with different configurations. These items were sunk to the bottom of the tanks and were chosen to stimulate the curiosity of the animals as novel objects and for tactile stimulation (rubbing on the PVC pipes). In order to stimulate foraging/hunting behaviors, turtles were provided with (iii) water cooler jugs with holes in the sides. Food items (fish and squid) were put into the jug, and it was sunk to the bottom of the tank. Bits of food would fall out of the jug as turtles pushed it across the bottom. The last ED (iv) was a waterfall, which was a water hose hanging over the side of the tank, above water, to allow water to splash into the tank. This was chosen as a novel and tactile enrichment. In the case of the blind turtle, it was observed that visual or olfactory enrichments would not be successful. Hence, the foraging/hunting device (cooler jug with holes) was replaced with a PVC lettuce feeder, which was an easier device to locate and manipulate, and the turtle was able to feel the food. The other ED used for the blind turtle was a carapace scratching device, as a direct tactile stimulation. A person placed in a particular part of the tank scratched the turtle carapace until the animal swam away. This enrichment imitated the action of fish or crabs cleaning the carapace of turtles [48]. The EDs had different effects on different animals. All four turtles exhibited a significant increase in random swimming and focused behavior and a significant decrease in pattern swimming and resting. This work showed that environmental enrichment can effectively increase curiosity and exploratory behaviors in captive marine reptiles, as it has been shown for other species. The application of enrichment has been associated with reduced aggressiveness or other undesirable behaviors in some individuals, reducing the captivity stress and increasing behavior complexity. This study showed that the blind turtle decreased stereotypical pattern swimming and increased other exploratory behaviors as it was provided with the appropriate enrichments. Positive behavioral changes were observed in all four turtles. They were more active when enrichment was present and spent less time resting during the day [48].

The second case used EE as a means to improve the rehabilitation of injured green sea turtles (*Chelonia mydas*) in Australia [53]. The EE in tanks was used to stimulate four sea turtles (unknown age and sex) presenting floating, with more natural behaviors to enhance their health and help speed recovery. The floating behavior indicated that turtles had special environmental needs. Thus, a series of EDs such as balls, pipes, boxes, brooms, food dispensing devices and a waterfall were used for each of the turtles for periods of 20 min. The same behavior categories used by [48] were employed in this study: resting pattern, repetitive pattern swimming, random swimming, focused behavior, orientation and non-categorized (not involved in a defined bahavior). Results showed a significant difference between time spent in the six different behaviors for all four turtles, and each individual behavior was significantly different from the others, depending on the enrichment device presented. It was observed that stereotypic behaviors such as pattern swimming or resting were reduced upon the presence of an ED [53].

The third case used EE in injured and long-time rehabilitated (10 years) loggerhead sea turtle *Caretta caretta* to determine its value to promote release and survival [54]. During rescue in 2006, it was observed that this turtle was severely wounded, showing injuries in both front flippers and missing nails in the hind flippers, and its body was covered in barnacles. This epibiont may be associated with hosting poor health such as immunosuppression and lethargy. This suggested that the turtle was probably entangled for a considerable time. After the turtle was released from the net, it was noticed that its right front flipper was severely damaged and required amputation of this limb at the level of the shoulder joint. The left flipper was also damaged but the wound did not affect the bone. This limb was saved after long therapeutic treatment using antibiotics and non-steroidal anti-inflammatory drugs [54]. The turtle was rehabilitated for over two years. Since it had a missing flipper, it was thought that this would restrict its successful release back to the wild. Due to the lack of information about the survival of three-flippered sea turtles in the wild, its release was not attempted for the next ten years. In 2016, the rescue center (CRAM) in Barcelona Spain, decided to release the turtle back to the wild. At the time, some data were available about the survival of turtles with missing limbs, such as sightings of nesting sea turtles with one or even two flippers missing over several years in Cape Verde. Also, the report of a loggerhead turtle with amputated fore flippers was released at the island of Cabrera, which was spotted a year later off the coast of Algeria in good health. In order to prepare the turtle to go back to the wild and to improve its chances of survival, a specific EE program was developed to enhance its species-specific behaviors. The EE program focused on three types of enrichment (nutritional, structural and sensory) with the objective to promote natural feeding behavior, to avoid man-made items (buoys) and to respond to environmental conditions [54].

In 2014, an EE program was implemented for six months using seven different EDs of the nutritional, sensory and structural types. These EDs were provided at random, and the animal reactions were recorded and evaluated. The turtle was submitted to a second EE program in 2016 before its release. This new program was developed in a pool of 4 m diameter, in an area separated from other sea turtles, sheltered and with reduced human contact. The turtle was acclimatized for four days to monitor and determine its basal response to the enclosure conditions such as usual diet, appetite, behavior (such as increased respiratory rate, rapid swimming, swimming against the wall of the pool, trying to climb out or prolonged immersions) and health condition. The EE was executed in a period of over two months when 14 different EDs were provided. Some days were ED-free, serving the usual diet or, alternatively, the turtle fasted. Daily, a different ED was presented at a random time of the day, and the responses to it were monitored using a webcam. Monitoring the reactions to EDs was completed by two persons, and the responses were evaluated by categorized descriptions of the expected reactions to the EDs. The evaluated reactions ranged from unfavorable (negative, neutral or not the expected reaction) to positive (initial interest but negative after 5 min, initial interest but neutral after 5 min, expected reaction but not during the appointed time and expected reaction) [54]. The EE program determined the most stimulating EDs for the animal. If an ED did not trigger the expected response, it was changed in order to ensure the expected behavior, especially feeding. The plan was flexible so these changes could be made one day in advance, minimizing the effect of negative responses to EDs. For example, if an ED caused the animal to escape, then no ED would be provided the next two days in order to get the animal back to a basal state. Along with the reactions monitored, blood samples were drawn once a month to determine stress hormone levels and the number of leukocytes during the EE program. These values would be compared to those from a database created in the 10 years the turtle was captive under rehabilitation [54].

The sea turtle responded as expected to all EDs. These included novel food items (sea urchins, crabs, jellyfish, different fish), man-made objects (buoys), wooden logs, rocks and artificial rain. The nutritional EDs were well accepted and consumed within the expected period of time from the beginning, including both live and dead items. The turtle distinguished and avoided man-made objects (buoys and boat defenses), but it did not evade natural objects such as a wooden log or rocks, which were used to scratch its carapace. Artificial rain had no effect on behavior. Blood analyses showed normal values. The weight of the animal increased slightly after two months under EE as well as its physical activity due to nutritional enrichment. These conditions promoted the decision to release the turtle. The animal was tracked with a satellite tag attached to the carapace to record its journey. After its prior location in the Mediterranean Coast off Catalunya, it was located at the strait of Gibraltar four months after release, when it swam to the Atlantic Ocean. The tag still emitted 10 months after release, when the animal reached the Azores and Madeira islands in the middle of the Atlantic Ocean, over 3500 km in a straight line from the release site. This case provided evidence that an impaired loggerhead turtle can be successfully released into the wild, even after being held in captivity for 10 years. The implementation of an EE program prior to release may have accounted for this success. This study supports the idea of releasing injured, disabled and/or confined turtles at rescue facilities for several years, despite the assumption that they may have reduced survival success [54].

The fourth case deals with the application of four EDs made of PVC to support a head-start program in the rearing of green turtle (*Chelonia mydas*) hatchlings before being released into their natural environment [55]. Hatchlings (15 d old) (*n* = 75) were distributed at random in 15 plastic tanks (3 tanks per treatment, 5 turtles per tank) and submitted to four different ED treatments and a control group (no exposure to any ED). Four items of PVC (ED) were used per tank. These had ring shape (RS), hollow square shape (HSQS), sphere shape (SS) and cylinder shape (CS). The outcome of each ED was compared to the control. Turtles were fed *ad libitum* twice daily with commercial pellets for carnivore fish, with 12:12 h light:darkness photoperiod and 100% water exchange daily before the first feeding [55].

The EDs were applied for 10 weeks and stayed in the tanks the whole day. They were removed from the experimental units only before water exchange. The effect of EDs on behavior was recorded from week 6 to the end of the experiment. Turtle activity was recorded once a week for 1.3 h using a digital camera just after water exchange when the EDs were returned into the tanks. The time (min/h) the animals engaged with the EDs was determined from the video, timed with a stopwatch and recorded. An interaction was determined if turtles performed any of these behaviors: (i) moved to touch the ED with its nose or flippers; (ii) the turtle’s neck moved in a vertical loop for a short time or the ED was placed on its carapace; or (iii) the animal perched on the device for ≥5 s. Resting on a device was not an interaction. Data of each turtle from the three tanks per treatment were analyzed as the mean for each tank. At the end of the experiment, the aggressive behavior of the turtles was assessed as the percentage of bite wounds on each of six areas of the body (head, neck, front flippers, hind flippers, carapace and tail). Data were scored for each turtle in the three tanks per treatment and presented as the mean per tank. No mortalities were recorded during the experiment and no differences between the growth or feed efficiency parameters were found. Hatchlings of the green turtle showed more interaction with the RS and then with the HSQS device. Less time was recorded interacting with the SS and CS devices. Nonetheless, the application of EDs significantly decreased the percentage of wounds in five out of the six areas monitored, with front flippers being the exception in the HSQS treatment. The most effective ED to hinder bite wounds was SS and then RS treatments, compared to the two other EDs and the control [46]. It was concluded that the RS was the most enriching ED for rearing green turtle hatchlings in captivity programs in Thailand, and this approach may be used in other zoos and aquaria. More research needs to be conducted to determine the enrichment effect of EDs against aggressive biting behavior in flippers and tails by changing ED design, such as other shapes, sizes and colors. This study supports the notion that using EE programs contribute to the welfare and wellbeing of green turtles reared in head-start programs in Thailand [55].

The fifth case is an EE program designed for one female olive ridley sea turtle (*Lepidochelys olivacea*) rescued and rehabilitated at the Centro de Rescate y Rehabilitacion de Animales Marinos (CRRAM) at the Pacific Marine Park, Puntarenas, Costa Rica. This turtle was rescued from net entanglement and its two front flippers had to be amputated due to the extensive damage presented. This turtle was in rehabilitation for two years before the EE experiment. In order to improve its welfare, the EE program was developed according to the results of a previous ethogram, which was used to record the number and types of activities or behavior displayed by the animal [56]. The turtle was observed daily for 1 h over five days in order to determine whether it displayed stereotypies such as resting, getting its head out of water to breathe (respiration) or displaying pattern swimming. Actions considered natural behaviors were recorded as random swimming and focused behavior. This basal ethogram was conducted one week before the start of the EE program (Table 1).

**Figure 1:**
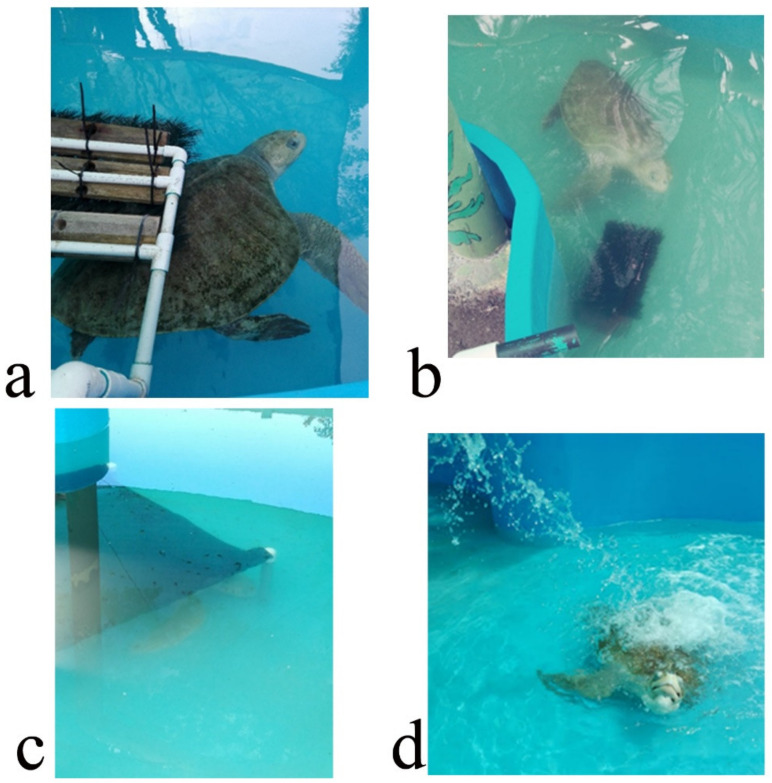
Environmental enrichment devices used in the rehabilitation of an olive ridley turtle (*Lepidochelys olivacea*). (**a**) carapace scratcher (sensory, tactile); (**b**) plastron scratcher (sensory, tactile); (**c**) shelter (structural, physical); (**d**) waterfall (structural, sensory, tactile).

**Figure 2:**
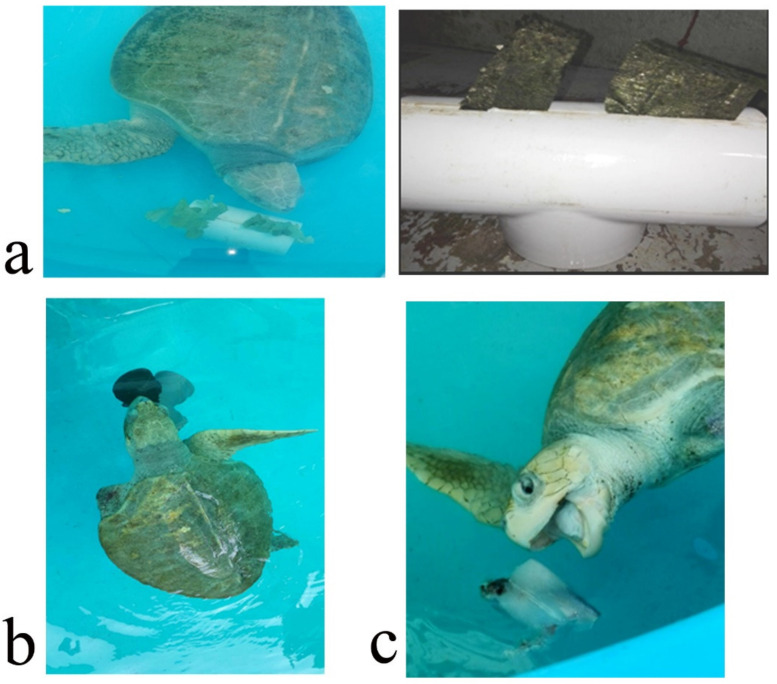
Environmental enrichment devices of the nutritional type used in the rehabilitation of an olive ridley turtle (*Lepidochelys olivacea*). (**a**) vegetable feeder to promote foraging; (**b**) feeder jar (nutritional, sensory); (**c**) ice block with food (nutritional, sensory).

The EE program was designed to increase its physical activity and to extend the amount of time used for feeding. It was intended to promote natural behaviors such as random swimming, hunting/foraging food items, seeking shelter and reducing boredom and stress. It was carried out for seven weeks, five days per week. The turtle was kept in a fiberglass tank of 4 m diameter. Seven EDs were separately applied, one per week, and the turtle was monitored for 1 h daily. It was monitored either in the morning or afternoon. Turtle activity was recorded by sight and the amount of time (min/h) it interacted with the ED was registered. The EDs were: (i) carapace scratcher (sensory, tactile) made up of PVC pipes and plastic bristles to allow the turtle to scratch and clean its shell and to promote relaxation; (ii) plastron scratcher (sensory, tactile), consisting of a plastic brush attached to a brick with rubber bands to allow the turtle to clean its plastron from algae, as well as to clean the ventral part of its flippers and tail. It also may help to avoid boredom and promote relaxation; (iii) shelter (structural, physical) was made from a piece of shade cloth attached to PVC pipes and placed in a specific part of the tank, intended as a refuge; (iv) waterfall (structural, sensory), which was a water hose placed on top in a side of the tank, making the water splash on the surface (Figure 1). This device was intended to avoid boredom and promote relaxation. The other three EDs were of the nutritional type in order to enhance natural feeding behaviors such as food-seeking, hunting and foraging to increase the time used for feeding and to promote curiosity. These consisted of: (v) vegetable feeder, made of PVC pipes with an incision where the algae, lettuce or spinach leaves were inserted. The aim was for the turtle to move the device to get the leaves out and eat them up, stimulating its foraging habits; (vi) feeding jar. This device was a small plastic water jar filled with chunks of squid or other shellfish. This gear was used to stimulate food-seeking behaviors; (vii) ice block. Chunks of shellfish (squid or fish) were frozen in water to produce an ice block containing the food items. The turtle had to play with or manipulate the ice block to thaw it and then eat up the shellfish. The aim was to increase the time used for feeding and to promote swimming (Figure 2). At the end of the EE, its efficacy was evaluated through an ethogram using the same elements as the basal ethogram. The level of turtle interaction with the EDs was determined using a categorical scale (none, some, plenty) (Table 2). The average time used by the turtle performing each behavior after the EE program is presented in Table 1.

Results showed that the EE program helped to improve turtle welfare. For instance, the time used for random swimming increased 17.5%, indicating that the turtle was far more active than before the EE. Resting behavior also diminished by 11.8%. It was noticed that the turtle used to rest more in the afternoon than in the morning. Respiration time was also reduced, since the turtle spent more time underwater, improving 5.1%. Under normal conditions, sea turtles are submerged for long periods of time. It was concluded that the EE program was successfully carried out and it helped improve turtle welfare. It was effective in stimulating normal behavior, physical activity and in reducing boredom and stereotypies such as resting and respiration, according to the previous data on the ethogram. The EDs used in the study boosted the curiosity of the turtle and helped to reduce fear and stress in the animal. The turtle used for the EE experiment has not been released into the wild and is still maintained at the facilities of the CRRAM.

## 6. Conclusions

Sea turtles are an important component of marine ecosystems worldwide, performing various ecological services such as regulators of communities, habitat engineering, sentinel and keystone species. Despite their importance, they are mainly threatened by anthropogenic activities. In order to save these species from extinction, various actions are required, including the rescue and rehabilitation of injured/diseased individuals. Animals rescued from the wild displaying major injuries, such as lost limbs, traumas and/or severe infectious or parasitic diseases derived from physiological imbalances, are considered true rehabilitated animals when they recover from such conditions and display near-normal behaviors prior to release into the environment. Conversely, sea turtles that were rescued and taken to rehabilitation centers in rather good health but sustained minor damages such as debilitation, dehydration or rescued from oil spills, are not considered to have undergone true rehabilitation [27,34].

Likewise, certain rescue practices such as taking eggs, hatchlings or adult turtles into captivity for care from their natural sites or environments for protection or safety, and later releasing them into nature at a location different from their original place, are considered translocations. The IUCN considers two translocation categories. The first one is called “Conservation translocation” and is defined as the deliberate movement of organisms from one site for release in another. This must provide a measurable conservation benefit at the population, species or ecosystem levels, and not be only for the benefit of the translocated individual. An example is the collection of turtle eggs from a nesting beach moved to a protected hatchery in another location from the same or another beach to be released in another beach. Conversely, category 2 involves the release of individuals for the sake of their welfare, or for rehabilitation from captivity, mainly for the benefit of the released individuals. An example is a translocation of rescued injured, ill or debilitated sea turtles that are then rehabilitated in captivity and later released into the environment. This action is probably a category 2 translocation because it has not been determined a measurable conservation benefit other than at the individual level [57].

Under captivity/rehabilitation, EE is a method that can successfully improve animal welfare and promote natural behaviors. EE may also help rehabilitated turtles with amputated flippers and/or long-time captive turtles to be successfully released back to the wild to perform their biological functions and probably to integrate into their natural populations, thus contributing to the conservation of the species. EE techniques may be implemented as complementary protocols in rescue and rehabilitation centers around the world to improve the welfare of captive/rehabilitating animals, thus contributing to enhancing conservation efforts for threatened or endangered species. Satellite tracking of released sea turtles may represent an effective way to monitor the success of rehabilitation practices in severely injured animals and the efficacy of conservation practices since it can record the length of survival, location, and behavior of rehabilitated individuals in the environment and within their populations.

## Figures and Tables

**Table 1:** Ethogram showing evaluated behaviors before and after environmental enrichment (EE).

	Before EE		After EE	
Features	Average		Average	
Behavior	min	%	min	%
Resting	32.7	54.5	25.6	42.7
Pattern swimming	0	0	0	0
Respiration	10.7	17.8	7.6	12.7
Random swimming	13.9	23.1	24.4	40.6
Focused behavior	2.7	4.6	2.4	4
Total	60	100	60	100

**Table 2:** Level of turtle interaction with the enrichment devices (ED) applied.

Enrichment Device (ED)	Time Applied (Days)	Level of Interaction		
		None	Some	Plenty
Carapace scratcher	5			X
Plastron scratcher	5			X
Shelter	5			X
Waterfall	5			X
Vegetable feeder	5		X	
Feeding jar	5			X
Ice block with food	5			X

## Data Availability

Not applicable.

## References

[1] Abreu-Grobois F.A., Gaona Pineda O., Barragán Rocha A.R. (2016). Generalidades de las tortugas marinas. Las Tortugas Marinas en México: Logros y Perspectivas para su Conservación.

[2] Sterling E.J., McFadden K.W., Holmes K.E., Vintinner E.C., Arengo F., Naro-Maciel E. (2013). Ecology and conservation of marine turtles in a central Pacific foraging ground. Chelonian Conserv. Biol..

[3] Cuevas-Flores E.A., Gaona Pineda O., Barragán Rocha A.R. (2016). Tortuga carey. Las Tortugas Marinas en México: Logros y Perspectivas para su Conservación.

[4] López Sánchez K., Gaona Pineda O., Barragán Rocha A.R. (2016). Tortuga laud. Las Tortugas Marinas en México: Logros y Perspectivas para su Conservación.

[5] Frazier J.G. (2005). Marine turtles: The role of flagship species in interactions between people and the sea. Mast.

[6] Aguirre A.A., Lutz P.L. (2004). Marine turtles as sentinels of ecosystem health: Is fibropapillomatosis an indicator?. EcoHealth.

[7] Bjorndal K.A., Bolten A.B. (2003). From ghosts to key species: Restoring sea turtle populations to fulfill their ecological roles. Mar. Turt. Newsl..

[8] Paladino F.V., Morreale S.J., Steele J.H. (2001). Sea turtles. Encyclopedia of Ocean Sciences.

[9] Ullmann J., Stachowitsch M. (2015). A critical review of the Mediterranean Sea turtle rescue network: A web looking for a weaver. Nat. Conserv..

[10] Innis C.J., Finn S., Kennedy A., Burgess E., Norton T., Manire C.A., Harms C. (2019). A summary of sea turtles released from rescue and rehabilitation programs in the United States, with observations on re-encounters. Chelonian Conserv. Biol..

[11] IUCN (2012). IUCN Red List Categories and Criteria: Version 3.1.

[12] WIDECAST Conservation Status/IUCN Red List. https://wwwwidecastorg/conservation.

[13] Norton T.M., Walsh M.T., Miller R.E., Fowler M. (2012). Chapter 31—Sea Turtle Rehabilitation. Fowler’s Zoo and Wild Animal Medicine.

[14] Mazaris A.D., Schofield G., Gkazinou C., Almpanidou V., Hays Graeme C. (2017). Global sea turtle conservation successes. Sci. Adv..

[15] Chaloupka M., Work T.M., Balazs G.H., Murakawa S.K.K., Morris R. (2008). Cause-specific temporal and spatial trends in green sea turtle strandings in the Hawaiian Archipelago (1982–2003). Mar. Biol..

[16] Flint M., Patterson-Kane J.C., Limpus C.J., Mills P.C. (2010). Health surveillance of stranded green turtles in southern Queensland, Australia (2006–2009): An epidemiological analysis of causes of disease and mortality. Ecohealth.

[17] Alwis S. (2020). Ecosystem Services of Sea Turtles. Scitubepro.com/ecosystem-services-of-sea-turtles.

[18] Koch V., Gaona Pineda O., Barragán Rocha A.R. (2016). Tortuga caguama. Las Tortugas Marinas en México: Logros y Perspectivas para su Conservación.

[19] Castro-Martínez M.A., Gaona Pineda O., Barragán Rocha A.R. (2016). Tortuga lora. Las Tortugas Marinas en México: Logros y Perspectivas para su Conservación.

[20] Domiciano G.I., Domit C., Bracarense R.L.A.P.F. (2017). The green turtle *Chelonia mydas* as a marine and coastal environmental sentinels: Anthropogenic activities and diseases. Ciências Agrárias.

[21] Bjorndal K.A., Jackson J.B.C., Lutz P.L., Musick J.A., Wyneken J. (2003). Roles of sea turtles in marine ecosystems: Reconstructing the past. The Biology of Sea Turtles.

[22] Gibson J., Smith G., Eckert K.L., Bjorndal K.A., Abreu Grobois F.A., Donnelly M. (1999). Reducing threats to foraging habitat. Research and Management Techniques for the Conservation of Sea Turtles.

[23] Steering Committee Bellagio Conference on Sea Turtles (2004). What Can Be Done to Restore Pacific Turtle Populations? The Bellagio Blueprint for Action on Pacific Sea Turtles.

[24] Putman N.F., Hawkins J., Gallaway B.J. (2020). Managing fisheries in a world with more sea turtles. Proc. R. Soc. B Biol. Sci..

[25] Regional Activity Centre for Specially Protected Areas (RAC/SPA) (2004). Guidelines to Improve the Involvement of Marine Rescue Centres for Marine Turtles.

[26] Marine Wildlife Watch of the Philippines (2014). Philippine Aquatic Wildlife Rescue and Response Manual Series: Marine Turtles.

[27] Robinson D.P., Hyland K., Beukes G., Vettan A., Mabadikate A., Jabado R.W., Rohner C.A., Pierce S.J., Baverstock W. (2021). Satellite tracking of rehabilitated sea turtles suggests a high rate of short-term survival following release. PLoS ONE.

[28] Delgado S., Nichols W.J. (2005). Saving sea turtles from the ground up: Awakening sea turtle conservation in North-Western Mexico. Mast.

[29] Cook M., Dunch V.S., Coleman A.T. (2020). An interview-based approach to assess angler practices and sea turtle captures on Mississippi fishing piers. Front. Mar. Sci..

[30] Foley A.M., Stacy B.A., Hardy R.F., Shea C.P., Minch K.E., Schroeder B.A. (2019). Characterizing watercraft-related mortality of sea turtles in Florida. J. Wildl. Manag..

[31] DuBois M.J., Putman N.F., Piacenza S.E. (2020). Hurricane frequency and intensity may decrease dispersal of Kemp’s ridley sea turtle hatchlings in the Gulf of Mexico. Front. Mar. Sci..

[32] DuBois M.J., Putman N.F., Piacenza S.E. (2021). A global assessment of the potential for ocean-driven transport in hatchling sea turtles. Water.

[33] Poloczanska E.S., Limpus C.J., Hays G.C., Sims D.W. (2009). Vulnerability of marine turtles to climate change. Advances in Marine Biology.

[34] Orós J., Montes de Oca N., Camacho M., Arencibia A., Calabuig P. (2016). Causes of stranding and mortality, and final disposition of loggerhead sea turtles (*Caretta caretta*) admitted to a wildlife rehabilitation center in Gran Canaria Island, Spain (1998–2014): A long-term retrospective study. PLoS ONE.

[35] Phelan S.M., Eckert K.L. (2006). Marine Turtle Trauma Response Procedures: A Field Guide.

[36] Mejía-Radillo R.Y., Zavala-Norzagaray A.A., Chavez-Medina J.A., Alonso-Aguirre A., Escobedo-Bonilla C.M. (2018). Presence of chelonid herpesvirus 5 (chhv5) in sea turtles in Northern Sinaloa, Mexico. Dis. Aquat. Organ..

[37] Whilde J., Whitmore L., Yang C., Eastman C.B., Thomas R., Rollinson D., Burkhalter B., Martindale M.Q., Duffy D.J. (2019). Behaviour of juvenile green turtles (*Chelonia mydas*) before and after fibropapillomatosis tumour removal. Testudo.

[38] Martínez-Silvestre A., Parga López M., Merchan Fornelino M. (2010). Capítulo 5. La recuperación clínica de las tortugas marinas. Tortugas Marinas de la Comunidad Valenciana: Conservación y Manejo Clínico.

[39] Herbst L.H., Eckert K.L., Bjorndal K.A., Abreu Grobois F.A., Donnelly M. (1999). Infectious diseases of marine turtles. Research and Management Techniques for the Conservation of Sea Turtles.

[40] Tracchia A.C. (2018). Medicina en Quelonios y Otros Reptiles.

[41] Bluvias J.E., Eckert K.L. (2010). Marine Turtle Trauma Response Procedures: A Husbandry Manual.

[42] Feck A.D., Hamann M. (2013). Effect of sea turtle rehabilitation centres in Queensland, Australia, on people’s perceptions of conservation. Endang. Species Res..

[43] Davis K.E. (2021). Compilation and Review of Sea Turtle Rehabilitation Protocols and Career Guidance from Conservation Professionals in the Eastern United States: Marine Science. Bachelor’s Thesis.

[44] Frazier J.G. (2014). La Situación Regional de las Tortugas Marinas en el Pacífico Sudeste.

[45] Whitherington B.E., Manire C.A., Norton T.M., Stacy B.A., Innis C.J., Harms C.A. (2017). Sea turtles in context: Their life history and conservation. Sea Turtle: Health & Rehabilitation.

[46] Mellen J., MacPhee M.S. (2001). Philosophy of environmental enrichment: Past, present, and future. Zoo Biol..

[47] Swaisgood R.R., Shepherdson D.J. (2005). Scientific approaches to enrichment and stereotypies in zoo animals: What’s been done and where should we go next?. Zoo Biol..

[48] Therrien C.L., Gaster L., Cunninham-Smith P., Manire C.A. (2007). Experimental evaluation of environmental enrichment of sea turtles. Zoo Biol..

[49] Cipreste C., Azevedo C., Young R., Kleiman D.G., Thompson K.V., Kirk Baer C. (2010). How to develop a zoo-based environmental enrichment program: Incorporating environmental enrichment into exhibits. Wild Mammals in Captivity: Principles and Techniques for Zoo Management.

[50] Maple T.L., Perdue B.M. (2013). Chapter 6. Environmental enrichment. Zoo Animal Welfare.

[51] Riley L.M., Rose P.E. (2020). Concepts, applications, uses and evaluation of environmental enrichment: Perceptions of zoo professionals. J. Zoo Aquar. Res..

[52] Vasconcellos D.S.A., Ades C. (2012). Possible limits and advances of environmental enrichment for wild animals. Rev. Etol..

[53] Lloyd J., Ariel E., Adams D., Owens L. (2012). Environmental enrichment for sea turtles in rehabilitation: Preliminary study. Proceedings of the 2012 Australian Wildlife Rehabilitation Council.

[54] Monreal-Pawlowsky T., Marco-Cabedo V., Palencia-Membrive G., Sanjosé J., Fuentes O., Jiménez E., Manteca X. (2017). Environmental enrichment facilitates release and survival of an injured loggerhead sea turtle (*Caretta caretta*) after ten years in captivity. J. Zoo Aquar. Res..

[55] Kanghae H., Thongprajukaew K., Inphrom S., Malawa S., Sandos P., Sotong P., Boonsuk K. (2021). Enrichment devices for green turtles (*Chelonia mydas*) reared in captivity programs. Zoo Biol..

[56] Bateson M.P. (2007). Measuring Behavior: An Introductory Guide.

[57] Caillouet C.W.J., Putnam N.F., Shaver D.J., Valverde R.A., Seney E.E., Lohmann K.J., Mansfield K.L., Gallaway B.J., Flanagan J.P., Godfrey M.H. (2016). A call for evaluation of the contribution made by rescue, resuscitation, rehabilitation, and release translocations to Kemp’s ridley sea turtle (*Lepidochelys kempii*) population recovery. Herpetol. Conserv. Biol..

